# Boosting Skin Cancer Classification: A Multi-Scale Attention and Ensemble Approach with Vision Transformers

**DOI:** 10.3390/s25082479

**Published:** 2025-04-15

**Authors:** Guang Yang, Suhuai Luo, Peter Greer

**Affiliations:** 1 School of Information and Physical Sciences, The University of Newcastle, Callaghan, NSW 2308, Australia; 2 School of Information and Physical Sciences, College of Engineering, Science and Environment, The University of Newcastle, Callaghan NSW 2308, Australia

**Keywords:** skin cancer classification, deep learning, transformer, image processing, neural networks

## Abstract

Skin cancer is a significant global health concern, with melanoma being the most dangerous form, responsible for the majority of skin cancer-related deaths. Early detection of skin cancer is critical, as it can drastically improve survival rates. While deep learning models have achieved impressive results in skin cancer classification, there remain challenges in accurately distinguishing between benign and malignant lesions. In this study, we introduce a novel multi-scale attention-based performance booster inspired by the Vision Transformer (ViT) architecture, which enhances the accuracy of both ViT and convolutional neural network (CNN) models. By leveraging attention maps to identify discriminative regions within skin lesion images, our method improves the models’ focus on diagnostically relevant areas. Additionally, we employ ensemble learning techniques to combine the outputs of several deep learning models using majority voting. Our skin cancer classifier, consisting of ViT and EfficientNet models, achieved a classification accuracy of 95.05% on the ISIC2018 dataset, outperforming individual models. The results demonstrate the effectiveness of integrating attention-based multi-scale learning and ensemble methods in skin cancer classification.

## 1. Introduction

Skin cancer is a prevalent medical condition, with over five million new cases diagnosed annually in the United States [1]. Australia experiences an even higher incidence rate per capita [2]. Melanoma, the most dangerous type of skin cancer, accounted for more than 190,000 new cases in the USA in 2019 [3], and it is responsible for the majority of skin cancer-related deaths. Late-stage melanoma diagnosis is associated with a poor prognosis [4]. However, when detected early, the survival rate can be as high as 97% [5], emphasizing the crucial need for early detection.

While melanoma is the most deadly form, benign skin cancers are more common. The resemblance between benign and malignant lesions complicates the accurate detection and classification of malignant skin cancers. Currently, dermatologists use dermoscopy, a high-resolution imaging technique, to aid skin cancer classification [6]. However, the reliability of diagnoses can be inconsistent with reliance on extensive clinical experience and visual expertise [7]. This variability points to the urgent requirement for more reliable classification methods to enhance early detection and improve patient outcomes.

Accurately classifying certain types of skin cancer, particularly malignant forms, has been a persistent challenge for both dermatologists and machine learning algorithms. To address this issue, deep learning techniques have been developed in recent years to enhance the accuracy of the diagnosis and classification of skin cancers. Recent advancements in deep learning have surpassed dermatologists in terms of accuracy and sensitivity [8]. However, for certain malignant skin cancers such as melanoma, even the most advanced algorithms still fall short due to the high degree of similarity between different lesion types.

To increase the accuracy of deep learning algorithms in skin cancer categorization, we conducted research to enhance these technologies. We took inspiration from the Vision Transformer (ViT) [9] and used its unique design to develop a performance booster that can be applied to any deep learning model. This method focusses on multi-scale learning and the detection of discriminative areas in pictures. Additionally, effective ensemble approaches were used to combine various models. These efforts resulted in high performance levels on the HAM10000 skin lesion database [10].

## 2. Related Work

Before the advent of deep learning in skin cancer classification, traditional machine learning techniques were pivotal in analyzing and classifying skin lesions. Researchers [11] utilized the ABCD (asymmetry, border, color, diameter) criteria as the key indicators for diagnosing skin lesions. These features were carefully analyzed to differentiate between benign and malignant lesions.

Various algorithms were employed for classification tasks. Support Vector Machines (SVMs) [12,13] were popular due to their effectiveness in handling high-dimensional data and robustness in classification tasks. SVMs work by finding the hyperplane that best separates the data into different classes. Additionally, clustering and classification methods such as K-means and K-nearest neighbor (KNN) algorithms were applied [14]. K-means partitions data into K-distinct clusters based on feature similarity, aiding in the preliminary grouping of similar lesion types. K-nearest neighbor (KNN), on the other hand, is a simple yet effective classification algorithm that assigns a class to a sample based on the majority class among its K-nearest neighbors in the feature space.

These traditional machine learning approaches laid the groundwork for more advanced techniques, providing valuable insights and establishing a baseline for accuracy and efficiency in skin cancer classification. However, with the introduction of deep learning, there has been a significant shift towards using neural networks and other sophisticated models that can automatically learn and extract features from raw data, leading to improved performance and accuracy in skin cancer classification.

Esteva et al. [8] presented a study in 2017 that compared the efficacy of skin cancer categorization using a pretrained GoogleNet Inception v3 model against dermatologists’ diagnosis. Since then, several researchers have contributed deep learning approaches to this subject, yielding many promising results.

In 2021, Datta et al. [15] achieved an accuracy of 93.4% on the HAM10000 dataset [10] with their innovative deep learning method. This approach integrated high-performing convolutional neural networks (CNNs) with soft attention layers. Additionally, they implemented a data preprocessing strategy that eliminated duplicate entries and balanced class distribution through both oversampling and undersampling techniques. The enhancement in model performance was significantly driven by the effective use of oversampling.

The Vision Transformer (ViT), which has inspired our methodology, was initially introduced in [9]. This groundbreaking work presented a model composed of multiple soft attention layers based on the transformer architecture originally developed for natural language processing (NLP). The ViT processes images in a manner similar to sentences in NLP transformers by dividing the input image into small patches and converting these patches into tokens. After pretraining on a large dataset and fine-tuning on a smaller dataset, the ViT demonstrated outstanding performance on several renowned benchmark datasets, including ImageNet.

Following the success of the Vision Transformer (ViT), researchers began applying it to skin cancer classification and segmentation from 2022 onwards. These studies focused on multi-scale learning and identifying discriminative image regions. In one study [16], researchers proposed a novel approach for image feature embedding, utilizing multi-scale and overlapping sliding windows to serialize images and perform multi-scale patch embedding. This method emphasizes capturing features at multiple scales. Another study [17] addressed the limitations of the original ViT by implementing an improved position encoding method that enhances multi-scale feature learning. Both studies achieved results comparable to those of previous state-of-the-art models in skin cancer classification.

In 2022, researchers [18] incorporated a spatial pyramid pooling module into the original transformer’s multi-head attention mechanism [19], enhancing computational efficiency and outperforming baseline convolutional neural network (CNN) models. Additionally, a recent study introduced FixCaps, which features a larger receptive field than that of Capsule Networks (CapsNets) by employing a high-performance large kernel at the bottom convolution layer. This study also integrated a convolutional block attention module to mitigate the loss of spatial information typically caused by convolution and pooling operations [20].

Recently, ensemble methods have gained popularity because they can significantly enhance predictive performance, even when the base models have similar performance levels. Several factors influence the extent of this improvement. Multiple studies [21,22,23] have explored various ensemble techniques, such as stacking and voting, to achieve better results. In our research, majority voting was applied to improve performance.

## 3. Our Method

**Figure 1:**
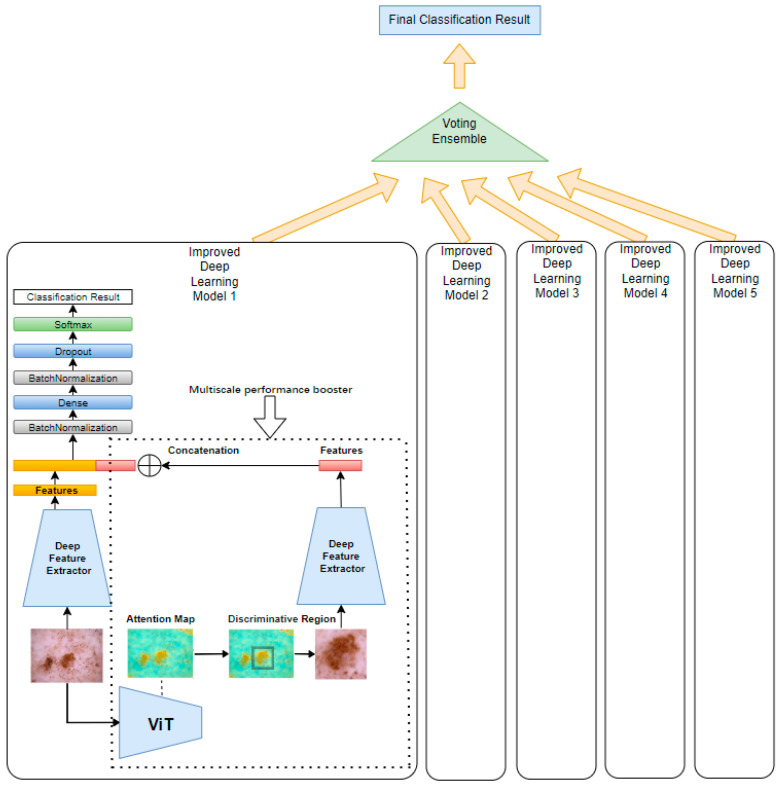
A multi-scale attention and ensemble approach with Vision Transformers.

Our proposed method, illustrated in Figure 1, begins by feeding a tumor image into a pretrained Vision Transformer (ViT). The ViT generates attention maps from the self-attention weights of each encoder block, where every image patch evaluates its relevance to others. By aggregating these attention weights across multiple layers and heads, we produce maps that highlight the most influential regions for classification—those most critical for accurate diagnosis. By applying a threshold to isolate these regions, these attention maps can be used effectively to segment the image into regions of interest, guiding the following model to focus on the most diagnostically relevant areas.

The method then implements a multi-scale analysis by cropping the highlighted regions to create zoomed-in views, capturing fine-grained details of the tumor. Simultaneously, both the original image and the cropped regions (zoomed-in with localized fine-grained details) are fed into two parallel, identical deep learning models. While the original image provides a broader context, the cropped images focus on more detailed features, offering a complementary perspective. This dual processing captures both global and localized information, making the model more sensitive to subtle patterns.

The performance gain is based on the principle that combining global and local features enhances the model’s ability to distinguish subtle patterns. The original image provides contextual information, while the cropped, high-attention region offers detailed, fine-grained features. By merging features at the extraction layer, we create a richer representation that emphasizes diagnostically relevant areas while suppressing irrelevant background information. This multi-scale fusion helps reduce noise and improves the model’s discriminative power, leading to higher accuracy and precision. By applying this multi-scale processing unit to both ViT and CNN models, we enhance their ability to segment and analyze tumors, acting as a performance booster that sharpens feature extraction. As shown in Figure 1, the same multi-scale analysis components and structure of model 1 were applied to models 2, 3, 4, and 5 with ViT Base B16, EfficientNetB2, EfficientNetB3, EfficientNetB4, and EfficientNetB5 to improve their performance.

To further amplify performance, an ensemble learning technique was applied through a majority voting mechanism, which aggregates predictions from multiple models. This ensemble approach enhances the robustness and reliability of the final prediction by leveraging diverse insights from various architectures, compensating for individual model biases and errors. Collectively, these innovations improve the accuracy and reliability of skin cancer classification.

In the following sections, a detailed description of our methodologies is provided, including the generation and application of attention maps, the construction and utilization of the model booster, and the implementation of ensemble learning techniques. These innovations collectively contribute to significant improvements in the accuracy and reliability of skin cancer classification.

### 3.1. Dataset Preparation

**Figure 2:**
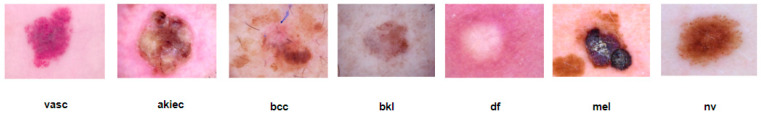
ISIC2018 image set samples.

In our experiment, the ISIC2018 [10] challenge dataset was used, also known as HAM10000. This dataset comprises 10,015 dermoscopic images, each with a resolution of 450 by 600 pixels. The images are categorized into seven distinct classes: actinic keratosis intraepithelial carcinoma (AKIEC), basal cell carcinoma (BCC), benign keratosis-like lesions (BKL), dermatofibroma (DF), melanoma (MEL), melanocytic nevi (NV), and vascular lesions (VASC), as illustrated in Figure 2.

The images in the ISIC2018 dataset are labeled by expert dermatologists and verified by histopathological diagnosis when available. Each image is annotated with one of seven classes based on clinical and pathological assessments:

- AKIEC (actinic keratosis and intraepithelial carcinoma): precancerous lesions characterized by irregular scaling, erythema, and atypical keratinocytes.
- BCC (basal cell carcinoma): the most common form of skin cancer, presenting as pearly papules with visible blood vessels (telangiectasia).
- BKL (benign keratosis-like lesions): these include seborrheic keratosis and solar lentigo; they are typically warty or have a stuck-on appearance.
- DF (dermatofibroma): benign nodules, often firm and hyperpigmented, typically located on the extremities.
- MEL (melanoma): malignant tumors arising from melanocytes; asymmetrical, irregular borders and varying color are common diagnostic cues.
- NV (melanocytic nevus): common benign moles; typically uniform in color and shape.
- VASC (vascular lesions): these includes angioma and hemorrhages; they are characterized by red or purple color due to blood vessel involvement.

To enhance classification performance, class rebalancing to the images in the training set was applied, which is crucial due to the inherent class imbalance in the dataset. The rebalancing process involved oversampling techniques to ensure that all classes have an equal number of images in the training set. Specifically, oversampling was applied to classes with fewer images using Keras data augmentation methods, which included random rotations up to 180 degrees, width and height shifts up to 10%, random zooming within a 10% range, and random horizontal and vertical flipping.

**Figure 3:**
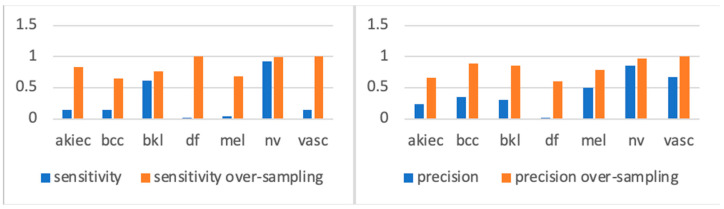
Sensitivity/precision comparison for each type trained with/without oversampling [24].

In a previous study [15], the author observed a significant improvement in model accuracy when trained on the oversampled version of the dataset, demonstrating the effectiveness of this approach. In another related study [24], researchers applied extensive class rebalancing, and the experimental results shown in Figure 3 demonstrated that training the same model on the rebalanced ISIC2018 dataset led to a significant performance improvement. We can see from the sensitivity figures in Figure 3 that, before oversampling, types with far fewer samples were underfitting compared with types with more samples (such as BKL and NV). However, after oversampling, each class acquired more balanced training. Accordingly, we used the oversampled ISIC2018 dataset for all our experiments.

### 3.2. Attention Map Generation Using Vision Transformer

#### 3.2.1. Vision Transformer (ViT) and Its Mechanism

The Vision Transformer (ViT) [9] is a deep learning model that adapts the transformer architecture [19], originally developed for natural language processing, for image classification tasks. Unlike traditional convolutional neural networks (CNNs), the ViT divides an input image into smaller, fixed-size patches, which are then flattened and embedded as vectors. These patch embeddings are treated as tokens, similar to how words are handled in NLP models.

#### 3.2.2. Transformer Encoder Blocks and Self-Attention

At the core of the ViT are transformer encoder blocks, each composed of multi-head self-attention and feed-forward layers. The self-attention mechanism allows each image patch to evaluate its relationship with other patches, effectively capturing both local and global dependencies. This capability is particularly useful for recognizing subtle patterns within images, such as identifying skin lesions.

Within each encoder block, the self-attention mechanism and feed-forward layers iteratively refine the patch representations. Residual connections and normalization layers are used to stabilize this process. By stacking multiple encoder blocks, the ViT can learn complex relationships across the entire image, ultimately enhancing classification accuracy.

#### 3.2.3. Classification Output

To aggregate information from all the patches, a special classification token ([CLS]) is added to the input sequence. After processing by the encoder blocks, the [CLS] token collects and integrates the information, which is then used for the final classification.

#### 3.2.4. Attention Maps in ViT

An important feature of the ViT is its ability to generate attention maps, which visualize the significance of different regions of the input image. The attention maps are graphical representations of the attention weights calculated between each token (i.e., patch) and all other tokens. Through the self-attention mechanism, each token assigns a weight to every other token, determining how much influence one patch should have over another.

These attention weights highlight the critical regions of an image that contribute most to the model’s understanding, making attention maps highly useful for interpreting model decisions. In applications such as skin cancer classification, attention maps can help identify and focus on the discriminative areas that are most relevant for accurately classifying skin lesions, thereby improving the model’s classification precision.

To construct the attention map, as illustrated in the original paper [9], attention weights from all layers of the transformer are aggregated to create a mask that highlights the object region while discarding irrelevant areas. This aggregation process is illustrated as follows: Suppose the transformer employs *H* heads, with *Q* and *K* representing the query and key vectors, respectively, each having a dimension of D. The attention weight AW is calculated using this formula:

(1) $$AttentionWeights=softmax(\frac{QK^{T}}{{\left(D/H\right)}^{1/2}})$$

If the head number *h* = 1, 2,…, H, the aggregated attention weights across multiple layers are calculated as follows:

(2) $$AttentionMap=\sum_{h=1}^{H}{AttentionWeights}_{h}$$

This process ensures that each token attends to every other token, focusing on the critical regions of the image. By aggregating these attention weights, we create a comprehensive mask that effectively emphasizes the object region, facilitating a more accurate and focused analysis of skin tumors.

**Figure 4:**
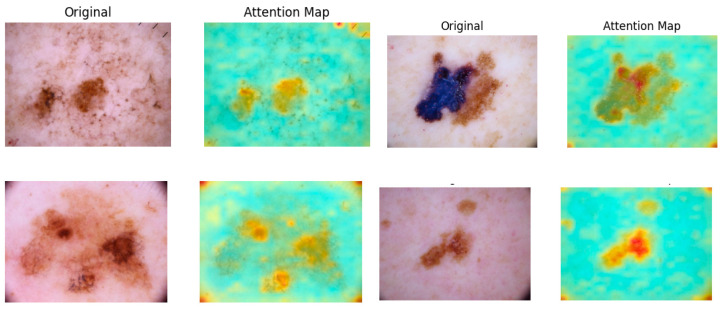
Attention maps generated in ViT for skin tumors.

As illustrated in Figure 4, attention maps are visualized as heatmap masks. In these visualizations, yellower colors signify higher attention weights between the respective tokens. By examining these maps, we obtain valuable insights into which parts of the image are most relevant for the classification task. This capability of segmentation is utilized in the next step to construct a multi-scale performance booster, as focusing on the most pertinent regions minimizes noise and improves classification precision.

### 3.3. Attention-Weight-Based Multi-Scale Performance Booster

After calculating the attention weights for the original image using a Vision Transformer (ViT), we proceed to calculate the mean pixel value of the attention map to establish a threshold. Pixels with higher values above this threshold are set to 1, while other pixels are set to 0, effectively creating a binary mask that highlights regions with higher attention weights.

**Figure 5:**
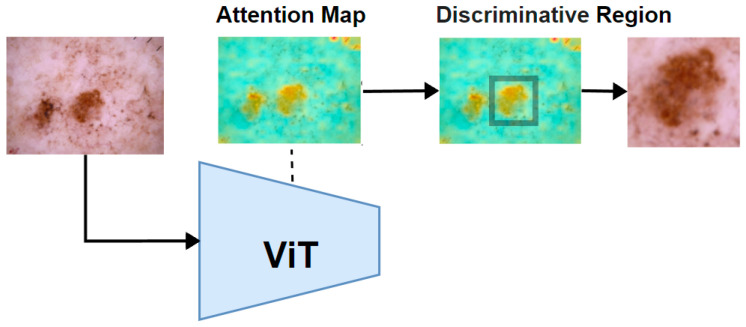
The attention map assists in identifying the discriminate region of the tumor.

Next, a traversal algorithm was applied to iterate through all the pixels in the binary mask to identify and isolate the largest connected region of high attention. Once this region is identified, we cropped the smallest enclosing rectangle around this area. This cropped region, which contained the most significant features as indicated by the ViT’s attention mechanism, was then used as the input for further feature extraction, as illustrated in Figure 5.

**Figure 6:**
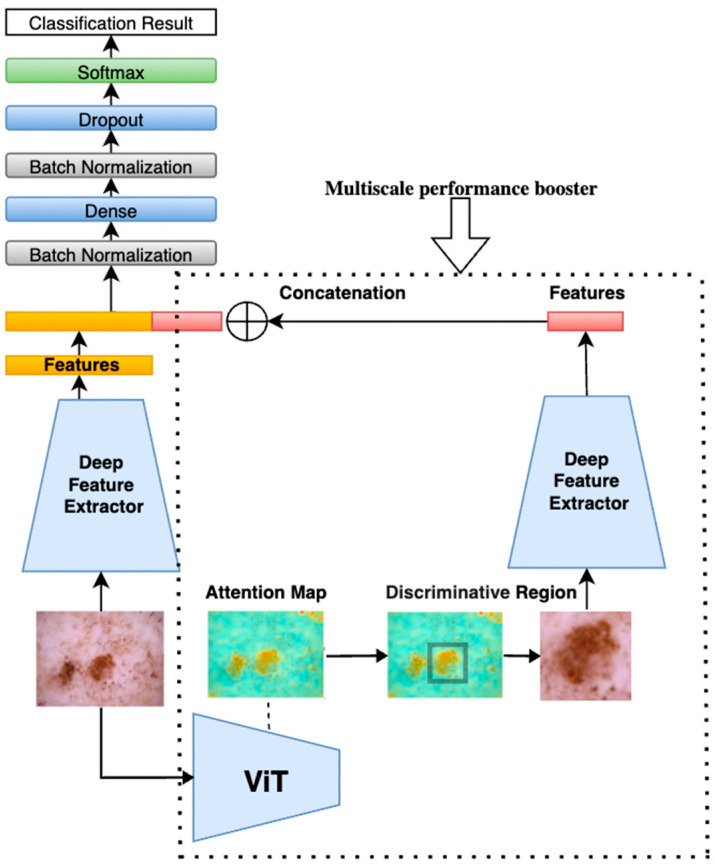
ViT-based multi-scale performance booster attached to a deep learning model.

After completing the previous step, two images were obtained: the original image and the discriminative region image. These images provide a multi-scale perspective of the tumor as shown in Figure 6. Both the original image and the cropped discriminative region image are then fed into two parallel yet identical deep feature extractors. While the original image provides a broader context, the cropped images focus on more detailed features, offering a complementary perspective. This dual processing captures both global and localized information, making the model more sensitive to subtle patterns. This setup ensures that features are extracted consistently from each image. The models independently extract features from each image, which are then combined by concatenating them at a flatten layer. This merging process creates a unified feature vector that incorporates information from both the original image and the cropped discriminative regions.

Following the flatten layer, a multilayer perceptron (MLP) is constructed, ending with a softmax layer to perform classification and complete the classification process.

Figure 6 illustrates the combined model structure. On the right side, inside the dotted rectangle, is the ViT-based multi-scale performance booster. This booster uses the same deep feature extractor as shown on the left side, ensuring consistent feature extraction across both components. In our experiments, both ViT models and CNN models were used, such as EfficientNet, for feature extraction, since ViT models demonstrated performance comparable to the latest CNNs. By incorporating the booster into these deep models, an accuracy improvement of 0.7–1.8% on the ISIC2018 dataset [10] was observed.

In this work, multi-scale refers to the use of both the original full-resolution image and a cropped region of interest (ROI) derived from attention maps. The original image provides a global view of the lesion and surrounding skin, while the cropped ROI offers a zoomed-in, high-detail representation of the most diagnostically relevant area. These two inputs capture different scales of information—global context and local detail—and are processed in parallel by identical deep learning models. The extracted features are later fused to form a unified representation. This multi-scale strategy enables the model to capture both a macro-level structure and micro-level texture, improving classification accuracy.

Additionally, while we use the mean pixel value as a global threshold to generate the binary attention mask, we acknowledge that this approach may not optimally adapt to the varying intensity and shape distributions of different lesion types. More adaptive thresholding methods, such as percentile-based selection or dynamic thresholding informed by lesion morphology, will be explored in future work.

### 3.4. Ensemble of Models

To further enhance the accuracy of our model, as shown in Figure 1, we employed a majority voting ensemble technique, which is a widely used method in machine learning. This method combined the predictions of multiple models to improve overall performance. Majority voting is defined as a decision-making technique in which multiple classifiers each make a prediction, and the final outcome is determined by selecting the option with the most votes. This helps improve model accuracy by combining multiple predictions. Each model “votes” for a specific outcome, and the outcome with the highest vote count becomes the final decision. This method leverages the diversity in predictions to enhance reliability and reduce overfitting, as the collective decision is often more robust than individual predictions.

This approach offers several advantages, including increased robustness, reduced variance, and the ability to leverage the strengths of different models. By aggregating the outputs of various models, the voting ensemble can provide more reliable and accurate predictions, especially in cases where individual models may have different strengths or make complementary errors. Research has shown that ensemble methods like voting can significantly boost the performance of machine learning models by fusing the informative knowledge obtained from multiple learning algorithms into a unified prediction [25].

In our ensemble, the outputs were combined of six different models: ViT Base B16, ViT Large L16, EfficientNetB2, EfficientNetB3, EfficientNetB4, and EfficientNetB5. The models selected are to ensure a balance between transformer-based and CNN-based architectures, as well as variation in model depth and computational complexity. ViT-B16 and ViT-L16 offer different transformer depths for capturing global dependencies. EfficientNetB2 through B5 provides multi-scale CNN feature representations across increasing input resolutions and capacities. To construct the voting ensemble, we first independently train each of the selected models—ViT Base B16, ViT Large L16, EfficientNetB2, B3, B4, B5—with each enhanced with the multi-scale attention-based performance booster. Once trained, these models generate individual predictions on the test set. For each test instance, the final classification is determined by majority voting, where the class label predicted by the majority of models is selected as the ensemble output. This diverse set of models allowed us to capture a wide range of features and patterns in the data, further boosting the accuracy of our predictions.

## 4. Experiment and Results

The experiments were conducted by first preparing the ISIC2018 dataset through a rigorous data cleaning process, followed by oversampling to address class imbalances. This ensured that each class had an adequate number of samples for effective training. Seven different models were trained individually, each enhanced with our attention-weight-based performance booster to improve their feature extraction capabilities and overall performance. After training these models separately, we combined their outputs using a voting ensemble technique. This approach allowed us to leverage the strengths of each model, leading to more accurate and robust predictions.

### 4.1. Data Preparation

The ISIC2018 dataset consists of 10,015 images across seven classes of skin disease. An 85/15 training/test split was applied to the original dataset. To maintain consistent class distribution between the training and test sets, we applied stratified sampling during the 85/15 split. This ensures that each skin lesion class is proportionally represented in both subsets, reducing potential evaluation bias, especially for minority classes. The dataset also underwent preprocessing, including duplication removal, class rebalancing, and pixel-level augmentation. Duplication removal eliminated duplicate images, and to prevent data leakage due to duplicate or near-duplicate lesion images in the ISIC2018 dataset, we used the lesion_id field to group images referring to the same lesion. Only one representative image per lesion_id was retained, and all duplicates were excluded prior to data splitting. This ensured that no duplicates appeared across both the training and test sets. Class rebalancing ensured an equal number of images per class in the training set to improve classification accuracy. Pixel-level augmentation involved operations like random saturation, contrast, and brightness adjustments. After these processes, the training set included a total of 52,692 images across the seven classes, with the number of images per class significantly increased through oversampling, as shown in Table 1.

### 4.2. Training Individual Models with Multi-Scale Performance Booster

In the individual training step for each model, we began by removing the classification head, converting the model into a feature extractor. This modification allows the model to focus solely on extracting meaningful features from the input data. Using the ViT attention map, we identified the discriminative region of the image, which highlights the areas most relevant to the classification task. This identified region was then fed into the feature extractor.

Simultaneously, the original, unaltered image was also fed into an identical feature extractor to ensure that the full context of the image was considered. Once the features were extracted from both the discriminative region and the original image, these two sets of features were concatenated to form a comprehensive feature representation. This combined feature set was then passed through a multilayer perceptron (MLP) to perform the final classification, allowing the model to make use of both the detailed discriminative region and the broader image context for improved accuracy.

All models were initialized with pretrained weights (e.g., ImageNet). Training was performed for 200 epochs using the Adam optimizer with an initial learning rate of 1 × 10^−4^, scheduled via cosine annealing. Batch sizes ranged from 4 to 16 depending on the model size and GPU memory availability. We used categorical cross-entropy as the loss function, appropriate for the multi-class classification task. An early stopping mechanism based on validation accuracy (patience of 10 epochs) was implemented to avoid overfitting. The dataset was split into 85% for training and 15% for testing, with 10% of the training set used for validation during training.

The overall accuracy improvements can be seen before and after applying the multi-scale performance booster from Table 2.

### 4.3. Ensemble

Applying majority voting, given the five models we have in the previous step, where *k* represents an individual model in the set {1, 2, 3, …, k}, we use the following formula to calculate the total accuracy of the ensemble model.

To calculate the total accuracy, the predictions of all models for each instance were aggregated, and the majority vote became the final prediction. The formula typically looks like

(3) $$Y=mode(y_{1},y_{2},\;\ldots ,\;y_{k})$$

where $y_{1}$, $y_{2}$, …, and $y_{k}$ represent the predictions of the individual models; the mode method refers to the statistical concept of the most frequently occurring value in a set of data; and *Y* is the final ensemble prediction, which is 95.05% (as shown in Table 3).

The tables below show the report of the classification of the final ensembled model.

In our experiment, accuracy, precision, recall, F1 score, and ROC-AUC were used to evaluate the performance of each model, of which the definitions are given below.

(4) $$Accuracy=\frac{True\;Positive+True\;Negative}{Total\;Number}$$

(5) $$Precision=\frac{True\;Positive}{True\;Positive+False\;Positive}$$

(6) $$Recall=\frac{True\;Positive}{True\;Positive+False\;Negative}$$

(7) $$F1\;Score=\frac{2*(Precision*Recall)}{Precision+Recall}$$

The ROC-AUC (Receiver Operating Characteristic Area Under the Curve) measures a model’s ability to distinguish between classes by plotting the True Positive Rate against the False Positive Rate at various thresholds. The models included in our ensemble operate at various input resolutions—from 260 × 260 (EfficientNetB2) to 456 × 456 (EfficientNetB5)—allowing for a diverse set of visual perspectives. While larger models operating at higher resolutions demonstrated only slight individual performance improvements, they were better suited for preserving fine-grained lesion characteristics such as irregular pigment patterns or blurred borders, which are particularly important in melanoma and AKIEC detection.

Notably, the ensemble significantly benefited from this resolution diversity. The combination of low-resolution models, which are more computationally efficient and effective at capturing broader context, with high-resolution models that focus on localized detail, enabled the ensemble to achieve superior accuracy. This multi-resolution feature fusion is especially valuable when handling a wide range of lesion sizes and visual complexities.

### 4.4. Comparison with Baseline Models and Recent Studies

To evaluate the effectiveness of our proposed multi-scale attention and ensemble strategy, we compared its overall classification accuracy on the same ISIC2018 dataset with the same setting using several baseline models and recent state-of-the-art approaches. As shown in Table 4, our method achieved an accuracy of 95.05%, outperforming classical CNNs (e.g., ResNet50), individual Vision Transformer variants (e.g., ViT-B16), and recent hybrid transformer-based models such as the Swin Transformer.

## 5. Discussion

### 5.1. Clinical Data Availability

In diagnosing suspected skin lesions, clinicians utilize a comprehensive approach that encompasses much more than basic visual assessments. A detailed review of the patient’s medical history, lifestyle factors, and clinical metadata are integral to this process. Elements such as exposure to sunlight, smoking habits, existing medical conditions, and current medications are evaluated alongside visual examinations and advanced imaging methods like dermoscopy to enhance diagnostic precision.

Critical diagnostic insights include the patient’s history of skin cancer, as it significantly influences the probability of recurrence. Additionally, factors like age; gender; ethnicity; and the anatomical position, dimensions, and morphology of the lesion are crucial. In cases involving hereditary conditions like melanoma, family history becomes particularly pertinent. The amalgamation of these diverse data points allows clinicians to develop a more comprehensive risk profile for the patient, thereby improving diagnostic accuracy.

Studies [26] indicate that equipping dermatologists—whether experienced or not—with this extensive clinical context markedly improves their diagnostic performance. Integrating detailed patient data helps clinicians make more informed decisions, potentially lowering the risk of misdiagnosis and improving patient outcomes.

Despite the evident benefits of incorporating clinical data, many initial studies on the application of deep learning (DL) for diagnosing skin diseases primarily focused on analyzing images of skin lesions in isolation. These studies often neglected the broader clinical context that human dermatologists rely upon. Consequently, the DL models developed under this paradigm may overlook critical information that could enhance their predictions.

The current challenge is to develop more advanced DL models that integrate both visual data and clinical context, replicating the multifactorial approach used by human dermatologists. By achieving this integration, we can bridge the gap between traditional diagnostic practices and AI-driven methodologies, ultimately resulting in more accurate and reliable outcomes. This holistic approach would not only improve the performance of DL models but would also ensure that AI systems are better aligned with the complexities inherent in real-world clinical decision-making.

In summary, integrating clinical data such as patient history and lesion metadata into DL models is crucial for enhancing their diagnostic capabilities and aligning them with the multifaceted approach used by clinicians. These improvements can lead to better patient outcomes and more accurate diagnoses.

### 5.2. Class-Level Performance Disparity

Despite the overall strong performance of our model, class-specific metrics reveal notable disparities, particularly in high-stakes categories. For example, the recall for melanoma detection remains relatively low at 0.71, suggesting that nearly 30% of melanoma cases may be misclassified—a significant concern in clinical applications. Similarly, the recall for AKIEC is 0.70, indicating reduced reliability in detecting early-stage carcinoma. These limitations are likely due to a combination of class imbalance, limited training samples for certain classes, and high visual similarity between malignant and benign lesions (e.g., melanoma vs. nevi, AKIEC vs. BKL). Improving recall for these diagnostically critical classes is a key priority for future work. Potential solutions include incorporating clinical metadata (such as lesion history and patient demographics), applying class-sensitive loss functions (e.g., focal loss), generating synthetic samples for low-performing classes, and leveraging multimodal data to enrich the feature space and reduce false negatives.

### 5.3. Model Generalization

Achieving model generalization remains a pivotal challenge in the realm of skin cancer classification. Predominantly, existing research has leaned heavily on medical imaging technologies like dermoscopy, which yield high-quality and standardized images. However, when these models are deployed on images captured with non-standard devices, such as smartphones or regular cameras, their performance tends to deteriorate significantly. This decline is primarily attributable to variations in image quality, lighting conditions, and other extrinsic factors that deviate from the controlled settings of medical imaging.

To mitigate this issue, transfer learning (TL) emerges as a potential solution, especially in the absence of a comprehensive training dataset specific to the new domain. TL facilitates the adaptation of models pretrained on extensive, well-annotated datasets from analogous domains to new tasks with limited data. By leveraging the pre-existing knowledge from related tasks, TL can help alleviate performance drops and enhance the generalization capability of models across diverse imaging modalities. Although researchers have made strides in this area by refining transfer learning techniques, there is still a need for extensive research to further bolster these capabilities. Some researchers [27] have contributed significantly to this domain by enhancing transfer learning, but continued efforts are essential to address the complexities involved.

### 5.4. Sampling Limitations and Attention Mislocalization

While our augmentation-based oversampling approach improved class balance and sensitivity for underrepresented categories, we acknowledge that such transformations (e.g., flipping, rotation, zooming) do not introduce true semantic diversity. The augmented samples, though visually varied, are still derived from the same original data, which may limit the model’s ability to generalize to unseen or rare lesion presentations. To mitigate this, we employed ensemble learning, which helped stabilize predictions and reduce overfitting by leveraging the strengths of multiple architectures.

Nonetheless, more sophisticated rebalancing strategies may be required to enhance robustness. In future work, we plan to explore methods such as synthetic data generation using GANs, mixup augmentation, and class-aware loss functions (e.g., focal loss or label smoothing) to improve representation learning for minority classes.

**Figure 7:**
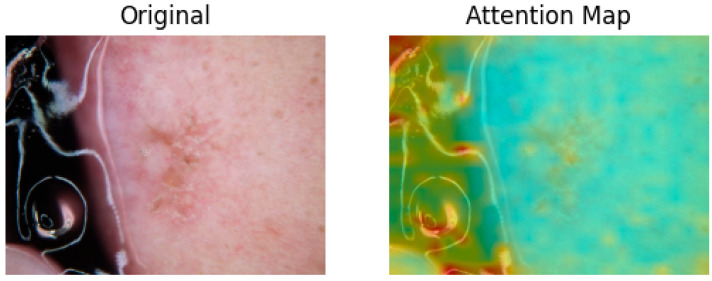
Attention map focusing on background objects for low-contract lesion.

Additionally, while the attention mechanism in our model generally helped to localize diagnostically relevant regions, we observed occasional failure cases where the attention maps were either diffuse or focused on irrelevant background areas. Figure 7 shows an example where the attention mask mistakenly highlights background objects instead of the actual lesion, likely due to low contrast or atypical lesion morphology. These cases highlight the limitations of our static thresholding method for generating attention masks, which does not adapt well across all lesion types. Future improvements may include dynamic thresholding, learnable attention refinement, or the integration of segmentation-guided attention to improve region-of-interest localization consistency.

**Figure 8:**
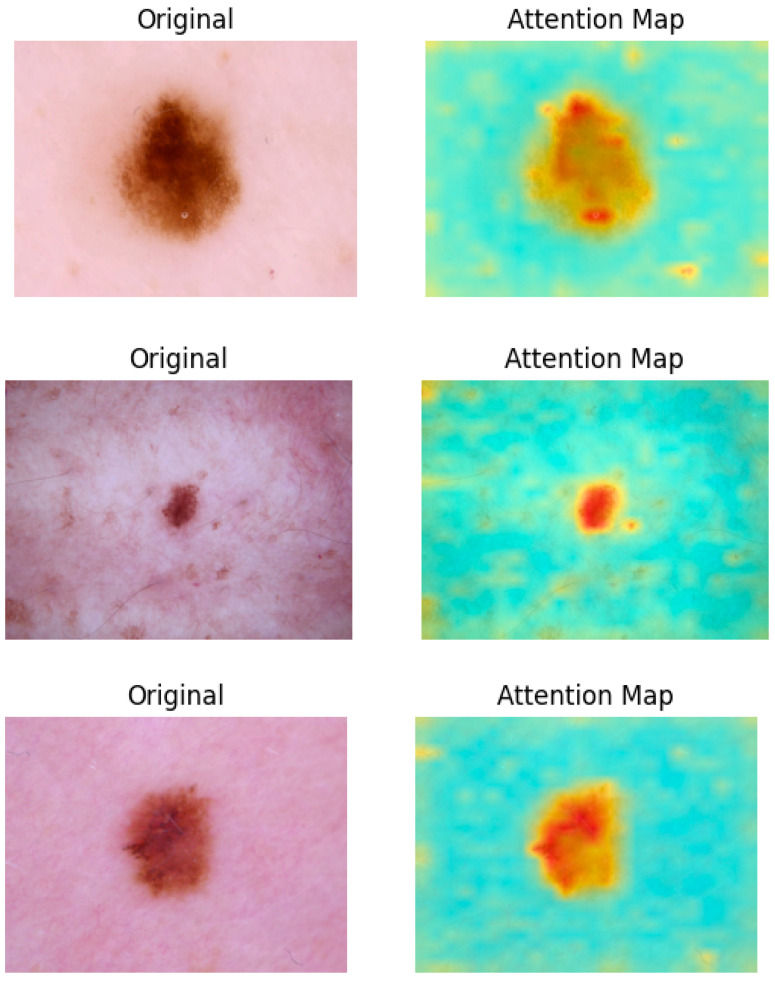
Some misclassified melanoma images.

To better understand the limitations of our model and the attention mechanism, we examined several misclassified melanoma images, particularly those that were wrongly predicted as benign nevus (NV). Figure 8 presents three such cases, each belonging to the melanoma class but classified as NV by the ensemble model.

These examples highlight the inherent challenge of early-stage melanoma diagnosis, where lesions often exhibit subtle or overlapping features with benign nevi. The first image (ISIC_0031529) shows a symmetric lesion with a relatively uniform color and smooth border—traits often associated with benign nevi. Similarly, the second (ISIC_0029209) and third (ISIC_0030818) cases display minimal asymmetry and lack clear structural irregularities that would be strongly indicative of malignancy. While our attention mechanism is designed to enhance the model’s sensitivity to subtle and fine-grained features, these particular lesions lack clear visual cues even for human assessment, making them extremely challenging to classify. In these cases, the attention maps still effectively focused on the lesion areas; however, the visual similarity to benign cases led the model toward incorrect predictions.

This analysis underscores the fact that even advanced attention-based models can struggle when lesions are visually ambiguous or underrepresented in training. To further enhance classification reliability in such cases, future work may explore integrating clinical metadata, increasing early-stage melanoma samples, and incorporating auxiliary supervision (e.g., segmentation masks or dermatopathology labels) to guide attention refinement in challenging diagnostic scenarios.

## 6. Conclusions

This study demonstrates the effectiveness of a multi-scale attention-based performance booster and ensemble methods in improving the classification accuracy of skin cancer using deep learning models. Leveraging the attention mechanism in Vision Transformers (ViTs), we identified discriminative regions within skin lesion images and combined this information with original image features, enhancing the models’ ability to differentiate between benign and malignant skin conditions. The application of our multi-scale booster significantly improved the performance of several state-of-the-art models, including EfficientNet and the ViT, as evidenced by an accuracy increase of up to 1.8% across various model architectures.

By integrating our ensemble approach and combining the predictions of multiple models through majority voting, the overall classification accuracy was further enhanced to 95.05% on the ISIC2018 dataset. The ensemble model’s performance in terms of the precision, recall, and F1-score across all seven skin cancer classes highlights its robustness and ability to generalize well across various skin lesion types.

In summary, this study presents a novel approach to enhancing skin cancer classification using a combination of ViT-based attention mechanisms and ensemble learning. The improvements observed in our experiments demonstrate the potential of these techniques in advancing the accuracy and reliability of AI-based skin cancer classification. Future work should focus on expanding the model’s generalization across different imaging devices and integrating clinical context to better align with real-world clinical practices.

## Figures and Tables

**Table 1:** Images in each class in training set after preprocessing.

Type of Skin Cancer	Number of Images	Images in Training Set After Preprocessing
AKIEC	327	6992
BCC	514	7858
BKL	1099	7931
DF	115	6876
MEL	1113	7903
NV	6705	8042
VASC	142	7090
Total	10,015	52,692

**Table 2:** Accuracy of models before and after applying multi-scale performance booster and ensemble learning.

Model	Configuration	Multi-Scale Booster	Ensemble	Accuracy (%)
ViT Base b16	Baseline	✗	✗	91.40%
	+ Multi-Scale Booster Only	✓	✗	92.63%
	+ Ensemble Only	✗	✓	93.50%
	+ Booster + Ensemble (Full)	✓	✓	95.05%
EfficientNetB2	Baseline	✗	✗	91.23%
	+ Multi-Scale Booster Only	✓	✗	92.39%
	+ Ensemble Only	✗	✓	93.50%
	+ Booster + Ensemble (Full)	✓	✓	95.05%
EfficientNetB3	Baseline	✗	✗	91.30%
	+ Multi-Scale Booster Only	✓	✗	92.63%
	+ Ensemble Only	✗	✓	93.50%
	+ Booster + Ensemble (Full)	✓	✓	95.05%
EfficientNetB4	Baseline	✗	✗	91.91%
	+ Multi-Scale Booster Only	✓	✗	93.72%
	+ Ensemble Only	✗	✓	93.50%
	+ Booster + Ensemble (Full)	✓	✓	95.05%
EfficientNetB5	Baseline	✗	✗	91.79%
	+ Multi-Scale Booster Only	✓	✗	92.51%
	+ Ensemble Only	✗	✓	93.50%
	+ Booster + Ensemble (Full)	✓	✓	95.05%

**Table 3:** Classification report.

Class	Precision	Recall	F1-Score	ROC-AUC
AKIEC	0.89	0.70	0.78	0.90
BCC	0.86	0.96	0.91	0.96
BKL	0.83	0.80	0.82	0.89
DF	0.83	0.83	0.83	0.92
MEL	0.83	0.71	0.76	0.85
NV	0.97	0.99	0.98	0.94
VASC	1.00	1.00	1.00	1.00
Accuracy			0.9505	
Macro-Avg	0.89	0.86	0.87	
Weighted Avg	0.95	0.95	0.95	

**Table 4:** Accuracy comparison.

Model	Dataset	Dataset Setting	Overall Accuracy
ResNet50	ISIC2018	Ours	0.905
ViT Base16	ISIC2018	Ours	0.914
ResNet50 + Attention (15)	ISIC2018	Ours	0.915
EfficientNetB5	ISIC2018	Ours	0.919
ViT Large16	ISIC2018	Ours	0.937
Swin Transformer	ISIC2018	Ours	0.931
Proposed Method	ISIC2018	Ours	0.951

## Data Availability

The original data presented in this study are openly available in the ISIC Archive at https://doi.org/10.7910/DVN/DBW86T (accessed on 1 September 2024).
